# Endoscopic transpalpebral approach for resection of an intraorbital intraconal cavernous angioma

**DOI:** 10.3171/2019.7.FocusVid.19168

**Published:** 2019-07-01

**Authors:** Matteo Zoli, Giacomo Sollini, Sofia Asioli, Clarissa Ann Elisabeth Gelmi, Angelo Gianluca Corradini, Ernesto Pasquini, Diego Mazzatenta

**Affiliations:** 1 Center for the Diagnosis and Treatment of Hypothalamic and Pituitary Diseases–Pituitary Unit, IRCCS Istituto delle Scienze Neurologiche di Bologna;; 2 Department of Biomedical and Neuromotor Sciences (DIBINEM), University of Bologna;; 3 ENT Department, Bellaria Hospital, Bologna; and; 4 Department of Biomedical and Neuromuscular Sciences, Section of Anatomic Pathology “M. Malpighi” at Bellaria Hospital, University of Bologna, Bologna, Italy

**Keywords:** orbit, endoscopic transpalpebral approach, cavernous angioma, histology, outcome, video

## Abstract

We present the case of a 47-year-old man with left exophthalmus. MRI showed a left intraorbital intraconal cavernous malformation, located in the superoesternal quadrant and medially displacing the optic nerve. An endoscopic transpalpebral approach was performed and total removal was achieved after dissection of the lesion from the optic nerve and other orbital structures. Pathology confirmed the diagnosis of cavernous malformation. The patient was discharged neurologically intact on the second postoperative day free of complications. Follow-up MRI demonstrated radical resection of the cavernoma and resolution of the exophthalmus with an excellent esthetic result.

The video can be found here: https://youtu.be/o1a1tneZ6qk.

## Transcript

### 0:20 Case presentation

This video represents the surgical management of an intraorbital intraconal cavernous angioma, located laterally to the optic nerve, in the left side.

A 47-year-old man presented with a 6-month history or progressive left exophthalmus. An MRI was performed, showing a spotting enhancing lesion mainly located in the superoexternal quadrant of the orbit, medially displacing the optic nerve, compatible for a cavernous angioma.

### 0:50 Discussion on possible surgical management

Many surgical approaches are possible for orbital lesions, mainly depending on their location, each one with proper advantages and limits (Martins et al., 2011; Paluzzi et al., 2015).

For lesions located anteriorly to the bulb, inferior or superior transconjunctival approaches may be adopted (Paluzzi et al., 2015). However, for deeper lesions other approaches are preferred, as the transcranial ones, which allow to enter into the orbit after its unroofing, such as for omolateral frontotemporal or frontotemporozygomatic or bifrontal (Khan and Varvares, 2006; Netuka et al., 2013; Liu et al., 2012).

The main limits of these routes are the brain retraction, required to expose the orbit roof, which could lead to possible postoperative complications, such as cerebral edema, stroke, seizures, and brain injury (Khan and Varvares, 2006; Netuka et al., 2013; Liu et al., 2012). Moreover, for tumors located in the inferior quadrants these approaches may require also a long corridor inside the orbit, with possible manipulation of the optic nerve or of its vascular supply (Khan and Varvares, 2006; Netuka et al., 2013; Liu et al., 2012).

The endoscopic endonasal approach may combine the advantages of not needing any brain retraction with a direct minimally invasive extracranial exposure of the lamina papiracea (Herman et al., 1999; Lee et al., 2012; Murchison et al., 2011). However, this approach is limited laterally by the optic nerve, which cannot be crossed to avoid permanent injury (Castelnuovo et al., 2015; Herman et al., 1999; Lee et al., 2012; Murchison et al., 2011).

Therefore, for tumors located lateral to this structure the alternatives to transcranial approach are represented by lateral orbitomy or other transfacial approaches, which are limited by the risk of not esthetic scars and which could require a careful reconstruction of the lateral wall of the orbit to avoid a poor cosmetic result for the patients (Castelnuovo et al., 2015). A promising option for such tumors, which has been recently proposed, is represented by the transpalpebral approach. It gives a direct and straightforward access to lateral quadrants of the orbit through a centimetric skin incision in the eyelid, which has not esthetic consequences (Dallan et al., 2016). Moreover, it allows to resect the lesion with the standard bimanual microsurgical technique with minimal manipulation of the orbital structures (Dallan et al., 2016).

### 3:04 Patient procedure

Our surgery was performed under general anesthesia with orotracheal intubation. Patient was positioned in simple supine position with head in a median position. A 2-cm linear incision was performed following a palpebral crease, starting from lateral canthus. The orbicularis oculi muscle was identified and dissected to start to identify the superoexternal orbital rim.

Periobita was dissected and retracted from this bone plane, which partially drilled out to increase the working space. The roof of the orbit was identified to orientate the surgeon.

Afterwards, periorbita was dissected and cut through a round scalpel and opened with scissors. With a hook this incision was enlarged and the orbital fat starts to come out.

Through a blunt dissection the intraorbital structures were progressively identified, mainly the lateral rectus muscles. During this phase it is important to avoid any manipulation of the ventral side of the muscles to avoid damages to neural motor fibers.

A large is mass was progressively identified medially to the lateral rectus muscle. The lesion was dissected from surrounding fat and other orbital tissue and isolated putting cottonoids at its sides. More tenacious fibers covering the lesion may be cut, after recheck of the position of the other anatomical structures.

During these dissection maneuvers, the dark-colored mass appeared in the surgical field. Its gross morphology was compatible with the preoperative suspect of cavernous angioma. Continuing with the blunt dissection of the lesions with cottonoids at its sides to avoid dangerous blind maneuvers and adopting sharp instruments only under the visual control of the surgeon on the front of the lesion, its anterior surface was now exposed and partially coagulated to shrink the mass.

With the same technique, it is possible to complete the dissection of the lesion, starting from its lateral side and then moving on the medial one, avoiding any direct traction or hazardous maneuver on the optic nerve, which was covered by cottonoids and not exposed.

Finally, the posterior portion of the cavernoma was resected with no direct manipulation of the lateral rectus muscle, which was anatomically and functionally spared, adopting the usual technique of dissection. Then, the tumor was delivered out of the orbit through the skin incision en bloc. Hemostasis was performed also adopting Floasel as direct hemostatic agent. A little piece of abdominal fat is placed to compensate the minimal lateral orbit wall drilling to avoid the development of enophthalmus.

### 6:55 Histological examination

Gross appearance of the mass was of a brownish lesion of about 2.5 cm of length. At histological examination, large, irregularly shaped and sized vascular channels surrounded by thick walls were observed without evidence of atypia. Endothelial cells showed positivity for antibody anti-CD 31, and they were negative for antibody anti-D2-40, confirming the diagnosis of cavernous angioma.

### 7:26 Follow-up

Patient was discharged intact at second postoperative day and MRI showed the complete resection of the tumor with resolution of exophthalmus. Cosmetic result was satisfactory with no evident scar on the superior eyelid.
